# Supplementary material for: Quantum memory with strong and controllable Rydberg-level interactions
Source: Nat Commun. 2016 Nov 21;7:13618. doi: 10.1038/ncomms13618 (PMC5121357; doi:10.1038/ncomms13618)
Supplement: Supplementary Information — Supplementary Figures 1-2 and Supplementary Notes 1-4. [file ncomms13618-s1.pdf]

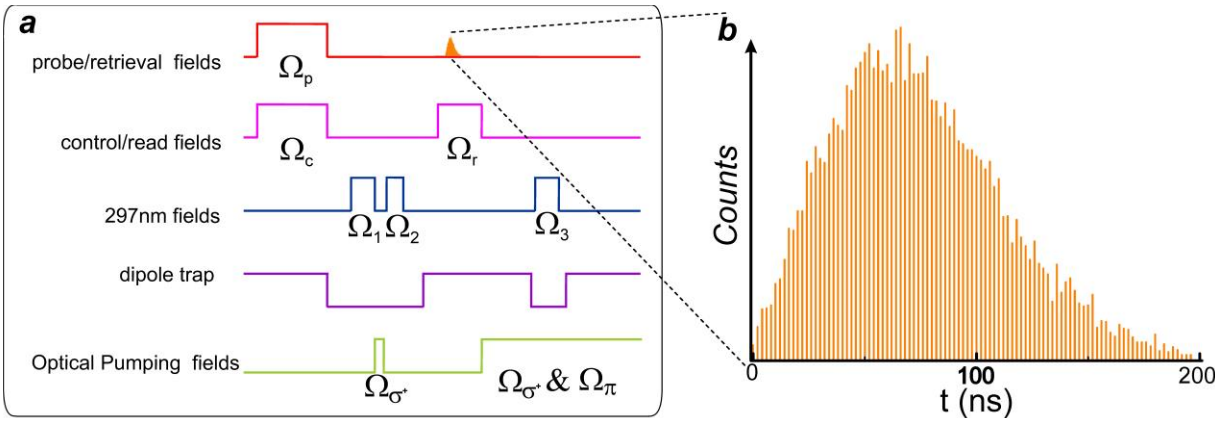

Supplementary Figure 1: **Timing protocol. a**, Experimental sequence of laser fields in the quantum memory protocol. **b**, Temporal profile of the retrieved field.

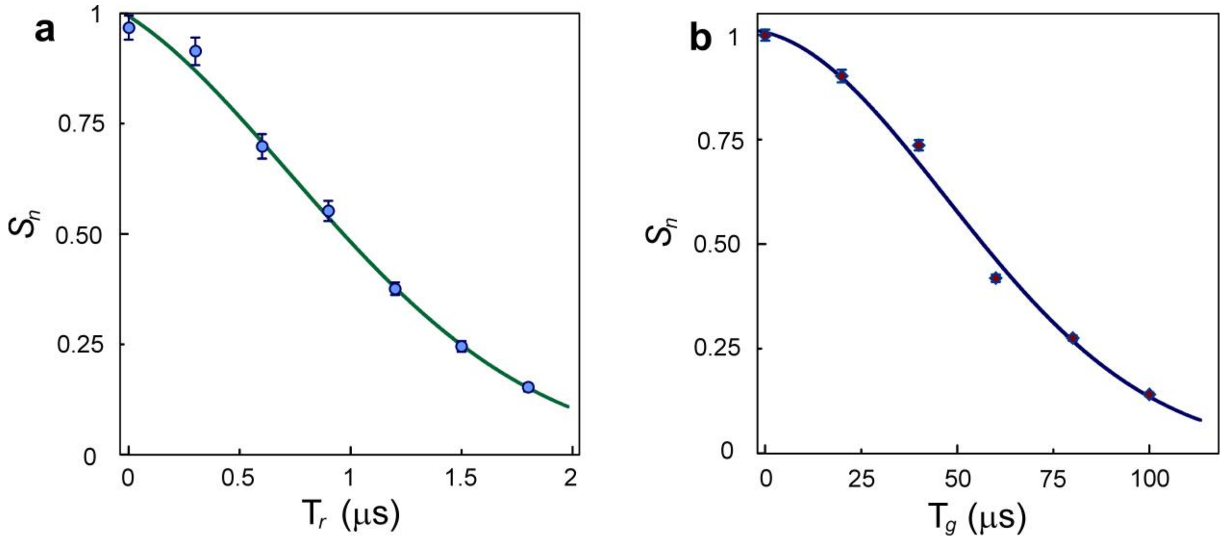

Supplementary Figure 2: **Atomic coherence properties. a**, Ground-Rydberg coherence time measurement, for the $|29p_{3/2}, m_J = -3/2>$ state. The normalized photoelectric detection rate S_n of the retrieved field is shown as a function of storage time T_r in the Rydberg state. A Gaussian function $\exp(-(T_r + T_d)^2/\tau_r^2)$ is used to fit the data, with $T_d = 0.4$ μs being a delay between two UV fields Ω_1 and Ω_2 for $T_r = 0$ and the best-fit value of $\tau_r = 1.58(2)$ μs. **b**, Coherence time measurement for the ground atomic states (Raman excitation only, no UV light applied). The rate S_n is shown as a function of storage time T_g. The data are fitted with the function $\exp(-(T_g + T_d)^2/\tau_g^2)$, with $T_d = 6$ μs being the delay between Raman excitation and read-out for $T_g = 0$, and the best-fit value of $\tau_g = 75(1)$ μs.

Supplementary Note 1. Experimental sequence.

Prior to execution of the quantum memory protocol, optical pumping is employed to prepare atoms in the ground state $|a\rangle = |5s_{1/2}, F = 1, m_F = 0>$. To empty the $F = 2$ hyperfine sub-level, we employ two laser fields: a π-polarized field Ω_π and a σ^+-polarized field $\Omega_{\sigma+}$. Both fields are resonant with the $|5s_{1/2}, F = 2> \leftrightarrow |5p_{1/2}, F = 2>$ transition. The Ω_π propagates along the x axis while the $\Omega_{\sigma+}$ field is mixed into the beam path of the control field Ω_c. To prepare atoms in the $|F = 1, m_F = 0>$ state, a π-polarized field Ω_{op} resonant with the $|5s_{1/2}, F = 1> \leftrightarrow |5p_{1/2}, F = 1>$ transition is used. After atoms are loaded and cooled in the dipole trap, alternating pulses of the Ω_{op} field and the $\Omega_\pi + \Omega_{\sigma+}$ fields are applied for 200 μs.

The 20-μs-long quantum memory protocol is repeated 8,000 times for each sample preparation. The overall duration of one experimental cycle is 0.78 s. Supplementary Figure 1 (a) shows the detailed timing sequence within each experimental protocol. To avoid dephasing the Rydberg-ground coherence by the differential optical trapping potential, the dipole trap is turned off after the 2 μs Raman excitation with Ω_{c} and Ω_{p} fields. 700 ns after the trap is turned off, Rydberg excitation and transfer fields Ω_1 and Ω_2 are applied. The dipole trap is turned back on and the read-out field Ω_2 converts ground excitations into approximately 200-ns-long light pulses (Supplementary Figure 1 (b)). The repeated application of the quantum memory protocol gradually re-populates the initially empty $|5s_{1/2}, F = 2\rangle$ levels, interfering with the Rydberg excitation process. To counter this effect, optical pumping fields Ω_π and $\Omega_{\sigma+}$ are applied. To remove the residual population in state $|b\rangle$ after the Rydberg excitation, a 200-ns-long $\Omega_{\sigma+}$ field is applied between Rydberg fields Ω_1 and Ω_2. After polariton retrieval, both Ω_π and $\Omega_{\sigma+}$ fields are turned on for 6 μs for further cleaning.

Supplementary Note 2. Rydberg excitation and read-out.

The 795 nm fields used to drive the Raman transition are derived from a pair of extended cavity diode lasers (ECDLs), each locked to a low-expansion, ultra-stable reference cavity. To reduce spontaneous emission from the intermediate state $|e> = |5p_{1/2}, F = 1, m_F = -1>$, each Raman field is detuned from the respective atomic resonance by a frequency offset $\delta_1/2\pi = -90$ MHz. The probe and control Raman fields have peak powers of 60 pW and 130 nW, respectively. The 297 nm UV light is derived by the fourth-harmonic

generation of an amplified 1188 nm laser light. The 1188 nm ECDL is frequency-locked to an ultra-stable reference cavity. The maximum power of 297 nm light on the atoms is about 20 mW.

The Rydberg transfer field Ω_2 and read-out field Ω_r have the same wave vector **k** as the Rydberg excitation field Ω_1 and the control field Ω_c, respectively. As a result, the retrieved field is approximately phase-matched into the spatial mode of the probe field Ω_p, which is coupled into a single mode fiber and split by a 50/50 fiber beamsplitter for the $g^{(2)}(\tau)$ measurement.

Supplementary Note 3. Preparation efficiency for single atomic excitations.

The probability of photoelectric detection P is proportional to the single excitation preparation efficiency ξ: $P = \eta_r \eta_{td} \xi$. Here η_r is the efficiency of converting a single excitation in state $|b\rangle$ into a retrieved-field photon. The photon transmission and detection efficiency $\eta_{td} = \eta_a \eta_f \eta_d = 0.24$, where $\eta_a = 0.75$, $\eta_f = 0.65$ and $\eta_d = 0.5$ are AOM diffraction efficiency, fiber coupling efficiency and single photon detection efficiency, respectively. We extract η_r from the overall efficiency of light storage $\eta_L = \eta_r \eta_s$, using the storage efficiency η_s determined from the transmitted fraction of probe field Ω_p. From the measured values of η_L = 0.00069(2) and η_s = 0.0111(2), we infer η_r = 0.062(2).

The efficiency of preparing single atomic excitations in state $|b\rangle$ can be determined as

$$\xi = P/(\eta_r \eta_{td}) = P \times 67(2). \quad (1)$$

Using the measured value of P = 0.12(1)%, we find $\xi \sim 8.1(6)$%. For the data shown in Figure 2(b) of the main text, the measured values of P (P_R) are normalized by η_r and η_{td} to obtain N (N_R). Here P and P_R are the probabilities of photoelectric detection with and without coupling to the Rydberg state, respectively.

For the interaction-induced dephasing mechanism, the efficiency of preparing a retrievable single excitation is limited by $1/e$. By employing Rydberg levels of higher principal quantum number n and/or smaller ensemble volumes, transition into the regime of Rydberg excitation blockade can be achieved, with a corresponding increase in preparation efficiency ξ. The latter is also affected by the (motional) Rydberg-ground decoherence, which can be mitigated by adopting a state-insensitive trap for ground and Rydberg atoms.

Supplementary Note 4. Coherence times and loss due to atomic diffusion.

The Rydberg-ground coherence for $|29p_{3/2}, m_J = -3/2>$ state is investigated in Supplementary Figure 2 (a). The coherence time $T_r = 1.58(2)\mu s$ is in agreement with the $1.58(5)\mu s$ measured coherence time for $|62p_{3/2}, m_J = -3/2>$. To study the coherence time of ground state levels, Raman excitation and retrieval are done without excitation to the Rydberg state. These data are shown in Supplementary Figure 2 (b).

During the preparation of quantum memory, the Gaussian profile of the probe field Ω_p results in a spatial density distribution of excitations in the transverse (x- and y-) directions. At zero delay between preparation and retrieval, the density distribution $n(x, T_g = 0)$ is

$$n(x, T_g = 0) = (\sqrt{2\pi\sigma_0 2})^{-1} \exp(-x^2/2\sigma_0^2), \tag{2}$$

where the cross-section radius $\sigma_0 = \omega_p/2$ and ω_p is the $1/e^2$ waist of the probe field. For an atomic cloud with temperature T, the atomic diffusion causes spatial broadening of $n(x)$ over time and leads to loss in retrievable excitations. The density distribution at a storage time T_g is given by

$$n(x, T_g) = (\sqrt{2\pi\sigma_0 2})^{-1} \exp(-x^2/2\sigma^2), \tag{3}$$

where $\sigma^2 = \sigma_0^2 + \sigma_v^2 T_g^2$, and $\sigma_v = \sqrt{kBT/M} \sim 0.03$ μm/μs. Considering the diffusion in x and y dimensions, the fraction of retrievable excitations at storage time T_g is given by

$$\begin{aligned} p(T_g) &= \frac{\int n(x, T_g) n(x, 0) dx \int n(y, T_g) n(y, 0) dy}{|\int n(x, 0) dx \int n(y, 0) dy|^2} \\ &= (1 + \frac{1}{2} {\sigma_v}^2 {T_g}^2 / {\sigma_0}^2)^{-1} \end{aligned} \tag{4}$$

To account for the difference between the measured $1/e$ lifetime of 71(2) μs in Figure 2(b) of the main text and the expected value of 80 μs from spin-wave dephasing, we fit the data while accounting for both spin-wave dephasing and diffusion loss and extract the $1/e^2$ transverse waist of the sample of $\omega = 6(1)$ μm, in agreement with the 5 μm $1/e^2$ waist of the probe field measured by the knife-edge method.
